# Vancomycin Insights: An Update on Mechanism, Activity, Toxicity, Resistance, and Novel Drug Delivery Systems

**DOI:** 10.5812/ijpr-160885

**Published:** 2025-09-02

**Authors:** Maryam Tabarzad, Maryam Torshabi, Mohadeseh Heidari, Azadeh Haeri, Seyedeh Maryam Mortazavi

**Affiliations:** 1 Protein Technology Research Center, Shahid Beheshti University of Medical Sciences, Tehran, Iran; 2 Department of Dental Biomaterials, School of Dentistry, Shahid Beheshti University of Medical Sciences, Tehran, Iran; 3 Department of Pharmaceutics and Pharmaceutical Nanotechnology, School of Pharmacy, Shahid Beheshti University of Medical Sciences, Tehran, Iran

**Keywords:** Vancomycin, Mechanism of Action, Toxicity, Resistance, Novel Delivery Systems, Liposomes, Nanoparticles

## Abstract

**Context:**

Vancomycin is an important antibiotic with a glycopeptide structure, effective against gram-positive bacterial infections through the inhibition of bacterial cell wall biosynthesis. It is indicated for the treatment of serious complicated skin, bloodstream, lower respiratory tract, bone, and joint infections. Primarily administered intravascularly, vancomycin shows negligible oral bioavailability. This review discusses the mechanism of action and toxicity of vancomycin, along with resistance issues and the mechanisms involved.

**Evidence Acquisition:**

We covered updated literature regarding various delivery systems for vancomycin, including nanoparticles, nanofibers, microparticles, liposomes, and hydrogels. Their antimicrobial evaluations and significant results are presented, alongside summaries of attempts for vancomycin oral delivery. The main issues of vancomycin, such as poor physicochemical properties (high molecular mass and aqueous solubility), poor oral bioavailability, minimum inhibitory concentration (MIC) creep, emergence of resistance, and high tendency to accumulate in the kidneys, can be addressed using novel drug delivery systems.

**Results and Conclusions:**

We summarized a range of novel drug delivery systems that have been investigated for enhancing the efficacy of vancomycin. The data collected here could be used as a guide for fabricating proper carriers for vancomycin delivery and selecting appropriate antimicrobial tests. Further in-depth studies on the mechanisms by which nanoparticles overcome resistance and enhance drug efficacy may pave the way for designing more effective systems.

## 1. Context

Vancomycin is an essential antibiotic with a branched tricyclic glycopeptide structure, first isolated in the 1950s from the soil bacterium *Streptomyces orientalis* in Borneo Island (1). It is effective against gram-positive bacterial infections by inhibiting the biosynthesis of bacterial cell wall peptidoglycan. Vancomycin is recommended intravenously for treating serious complicated skin, bloodstream, lower respiratory tract, bone, and joint infections, as well as endocarditis and meningitis. It is particularly used for severe infections caused by methicillin-resistant bacteria, such as *Staphylococcus aureus* (MRSA) and *Staphylococcus epidermidis* (MRSE), endocarditis caused by resistant staphylococci, highly penicillin-resistant meningitis caused by *S. pneumoniae*, and pneumonia caused by penicillin-resistant *S. pneumoniae* (1, 2). The usual adult dose is 30 mg/kg/day, divided into 2 or 3 smaller doses (2). Oral vancomycin is indicated for the treatment of MRSA-associated enterocolitis and Clostridium difficile-mediated diarrhea (3). Its oral administration achieves high gastrointestinal levels without systemic absorption (1).

**Figure 1.**
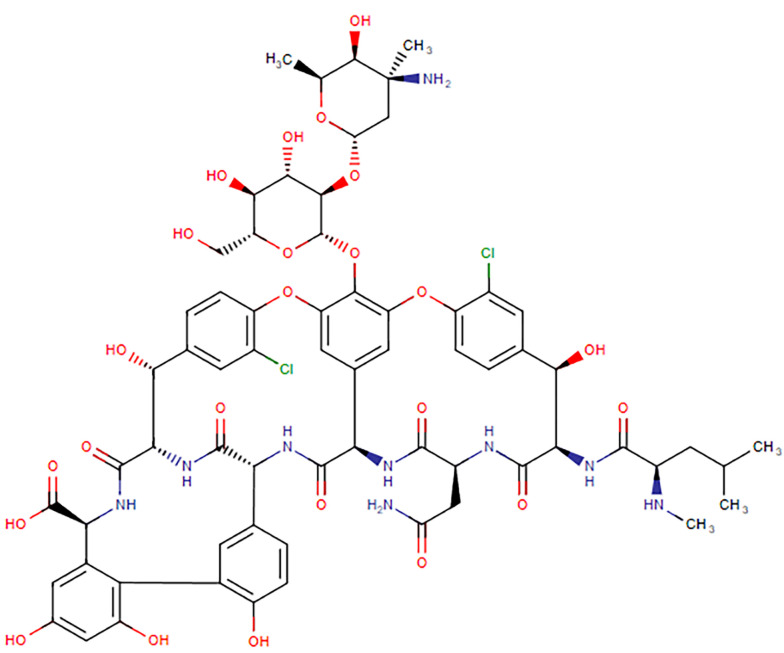
Chemical structure of vancomycin (3)

Vancomycin (C66H75Cl2N9O24, Figure 1) has a molar mass of 1449 Da and is a hydrophilic compound. Its physical properties include a water solubility of 0.225 mg/mL and an experimental log P of -3.1 (3). Oral absorption is limited due to degradation in the acidic gastric environment, enzymatic degradation, and low intestinal permeability (4). It belongs to the Biopharmaceutical Classification System (BCS) class III drugs, characterized by high aqueous solubility and low intestinal permeability (5, 6). Vancomycin's volume of distribution and protein-binding are reported to be 0.4 - 1 L/kg and 10 - 50%, respectively. It is primarily eliminated through the kidneys, with more than 80% of the dose recovered in urine during the first 24 hours post-administration. The drug's half-life is approximately 6 hours in patients with normal renal function (2, 7).

The clinical importance of vancomycin has been well established. However, serious adverse effects such as nephrotoxicity, "red man" syndrome, allergic reactions, and other side effects (1) are reported. Patients with pneumonia, endocarditis, and osteomyelitis are more prone to acute kidney injury (AKI) and chronic kidney disease (8). In addition to systemic toxicity, the first isolates of vancomycin-resistant Enterococci (VRE), namely *Enterococcus faecalis* and *Enterococcus faecium*, have emerged after 30 years of successful application to treat severe infections. Over time, the resistance property of this drug has been transferred to other bacteria, including *S. aureus*. Therefore, the rapid rate of resistance emergence to vancomycin has become a clinical concern, leaving some patients infected with staphylococci and enterococci with few therapeutic options (9, 10).

**Figure 2.**
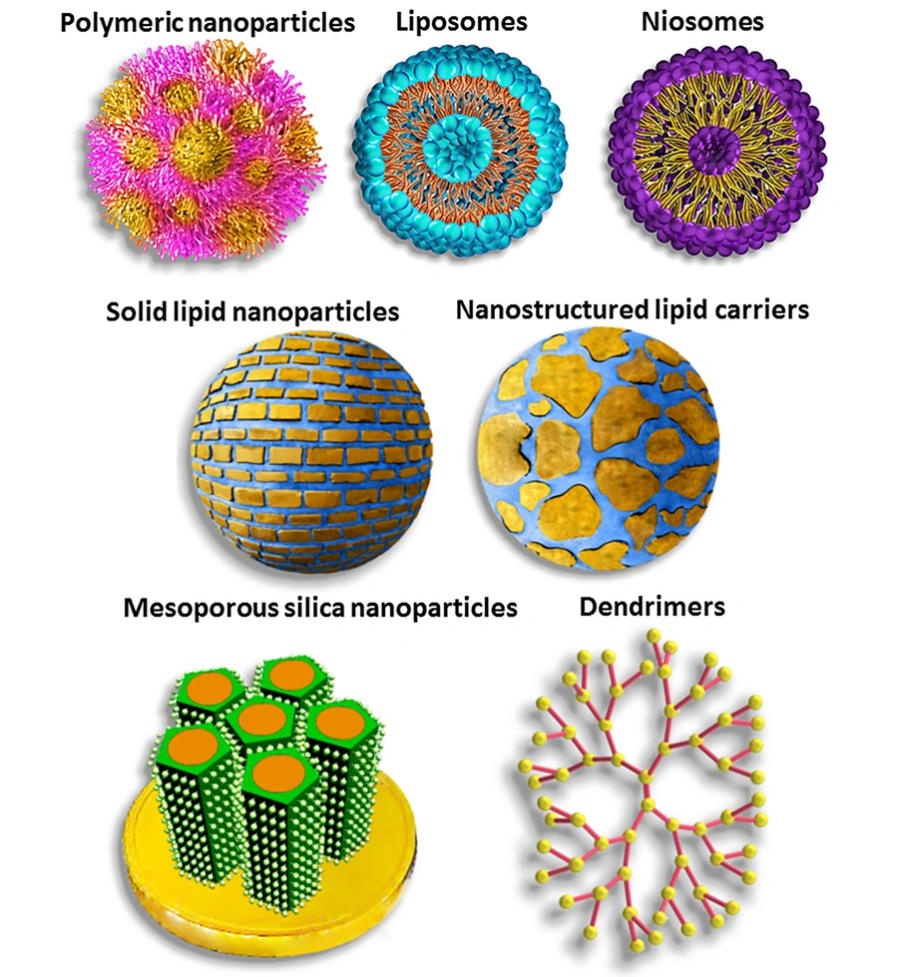
Schematic structures of different nanocarriers used for vancomycin delivery [reprinted with minor modification from Ref. (13] with permission)

To address this issue, new generations of antibiotics (9) as well as novel delivery systems (11) have been developed. Carrier-mediated delivery employing nanostructures (Figure 2), microstructures, and hydrogels provides a promising strategy to deliver drugs to the site of action, reduce off-target levels, minimize systemic toxicity, enhance biological barrier transport, improve drug pharmacokinetics and pharmacodynamics, provide controlled and on-demand drug release, and improve interaction with microorganisms (11-15). However, vancomycin’s hydrophilicity and high molecular mass make designing proper delivery systems challenging. In this review, we discussed the mechanism of action and toxicity of vancomycin. Moreover, resistance issues and the involved mechanisms were covered. Polymeric nanoparticles (16, 17), liposomes (18, 19), nanofibers (20), dendrimers (21), metal nanoparticles (22), microparticles (23), hydrogels (24), and many others have been investigated for vancomycin delivery, which will be discussed in this context.

## 2. Mechanism of Action

Vancomycin binds to the D-Ala-D-Ala terminus of Lipid II, a key component in the synthesis of the cell wall, inhibiting peptidoglycan synthesis, which compromises bacterial cell integrity (25). Vancomycin prevents the cross-linking of peptidoglycan strands, resulting in an incomplete and defective cell wall structure. This disruption compromises the structural integrity of the bacteria, making them susceptible to osmotic pressure and ultimately resulting in cell lysis and death (26). In addition to inhibiting cell wall synthesis, some vancomycin conjugates could alter the permeability of the bacterial cell membrane, leading to further disruption of cellular processes (27).

The vancomycin-Lipid II complex is stabilized by hydrogen bonds and hydrophobic interactions with the D-Ala-D-Ala moiety. This specific binding is crucial for its effectiveness (28). Some studies have suggested that vancomycin may also inhibit RNA synthesis within bacteria, although this effect is less well characterized compared to its primary action on cell wall synthesis. In an initial report, Jordan and Inniss (1959) observed that vancomycin inhibited RNA synthesis in growing cultures of *S. aureus*, while not affecting DNA and protein production (29). Further research confirmed that vancomycin primarily inhibited the synthesis of cell wall mucopeptides in *S. aureus*, occurring within 2 to 5 minutes of exposure. The inhibition of RNA synthesis followed at about 20 minutes, with a slight impact on DNA synthesis after 30 minutes. Additionally, vancomycin affects the uptake of C14-labeled amino acids, indicating broader effects on polypeptide synthesis. The observed RNA synthesis inhibition is likely a secondary effect stemming from the initial blockage of mucopeptide production (30).

**Figure 3.**
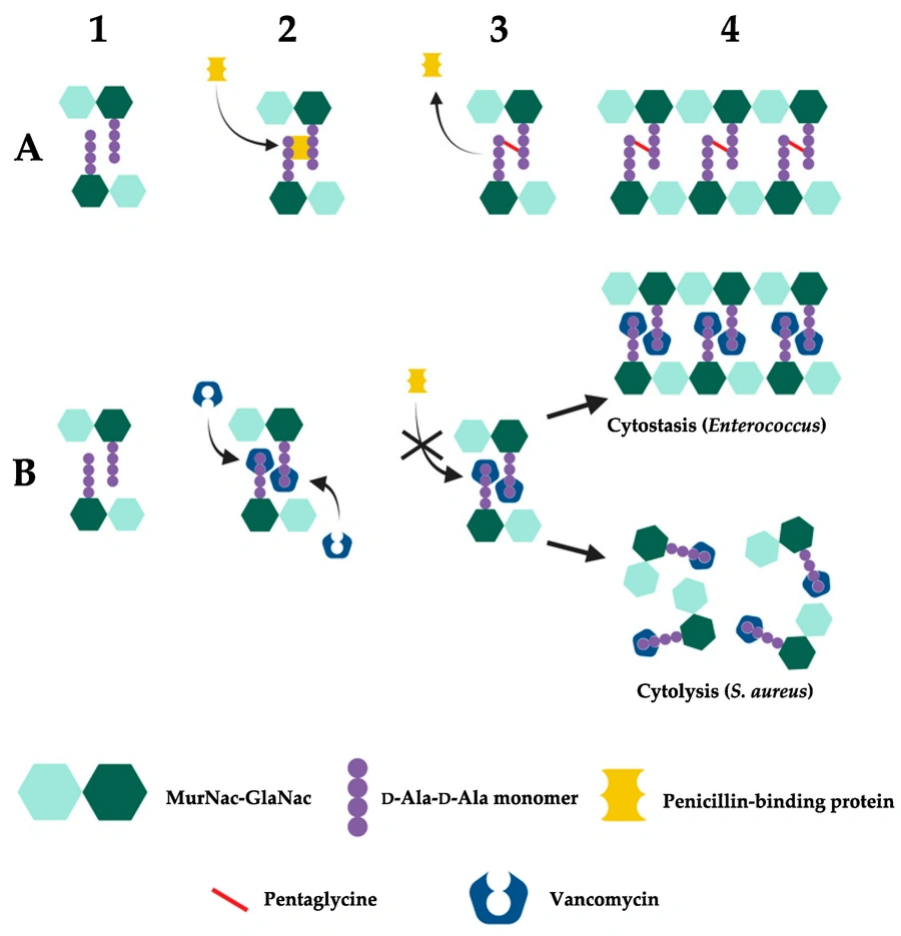
Mechanism of vancomycin action: A, in susceptible bacteria, normal cell wall synthesis occurs through enzymatic cross-linking when vancomycin is absent. The number 1 to 4 presents the process of peptidoglycan synthesis from D-Ala-D-Ala monomers. Penicillin-binding proteins (PBPs) recognize and bind to the monomers and promote the cross-linking of the peptidoglycan monomers; B, vancomycin binds to D-Ala-D-Ala monomers and inhibits the cross-linking of peptidoglycan in susceptible bacteria. It prevents PBPs from catalyzing pentaglycine bond formation (31) (reprinted from an open access journal under the Creative Commons CC-BY license: https://creativecommons.org/licenses/by/4.0/).

Figure 3 schematically presents the mechanisms of action of vancomycin. In bacteria, normal cell wall synthesis occurs through enzymatic cross-linking involving transglycosylase and transpeptidase when vancomycin is absent. In this condition, the peptidoglycan structure starts with unlinked D-Ala-D-Ala monomers. Then, penicillin-binding proteins (PBPs) recognize and bind to the D-Ala-D-Ala monomers, promoting the cross-linking of the peptidoglycan D-Ala-D-Ala monomers by catalyzing the formation of pentaglycine bonds. Finally, a newly formed cell wall with fully cross-linked D-Ala-D-Ala monomers is generated. However, vancomycin inhibits the cross-linking of peptidoglycan in susceptible bacteria by binding to D-Ala-D-Ala monomers. First, vancomycin binds to the D-Ala-D-Ala monomers. Then, vancomycin's binding prevents PBPs from catalyzing pentaglycine bond formation. As a result, the cross-linking of peptidoglycan is blocked, disrupting cell wall synthesis, which can lead to cellular stasis in Enterococcus or cell death in *S. aureus* (31).

## 3. Toxicity Mechanisms of Vancomycin

Vancomycin has shown a range of adverse effects, from allergic reactions and infusion-related symptoms to severe nephrotoxicity and ototoxicity (2). Nephrotoxicity is a significant concern with vancomycin use, affecting up to 40% of patients, especially with high doses or prolonged therapy. Acute interstitial nephritis (AIN) and acute tubular necrosis (ATN) are noted mechanisms of renal injury, necessitating careful monitoring of renal function during treatment (32).

In a study of 36 patients who underwent renal biopsies for AKI suspected to be caused by vancomycin, researchers identified 25 patients with vancomycin nephrotoxicity (VNT) and 11 without nephrotoxicity (NO-VNT). The VNT group exhibited a distinct clinical profile characterized by high serum trough levels of vancomycin, rapid and severe AKI, and recovery of renal function following discontinuation of the drug. Renal biopsies revealed acute tubulointerstitial nephritis (ATIN), granulomatous inflammation, ATN, and vancomycin casts, unlike the NO-VNT group (33).

From a review of VNT, it was found that pathological manifestations include ATN, ATIN, and intratubular crystal obstruction, with proposed mechanisms involving oxidative stress, allergic reactions, and vancomycin-associated tubular casts (VTCs). Factors such as the concurrent use of other nephrotoxic antibiotics, high doses of vancomycin, and dosing strategies (intermittent vs. continuous infusion) elevate AKI risk. While several biomarkers exist for predicting and diagnosing AKI, no effective therapies are currently available; oral steroids may help ATIN patients, and hemodialysis can remove vancomycin (34).

Oxidative stress, resulting from the imbalance between reactive oxygen species (ROS) and antioxidants inside cells, is a key factor in vancomycin-associated acute kidney injury (VA-AKI). When vancomycin is taken up by proximal tubular cells, it triggers oxidative phosphorylation, increasing ROS production and leading to mitochondrial dysfunction, ultimately causing cell death. Research indicates that a higher ratio of 5-hydroxy indole acetic acid (5-HIAA) to serotonin (5-HT) could potentially serve as a biomarker for VA-AKI, reflecting acute oxidative stress and inflammation. The increased ROS activates genes related to oxidative stress, causing lipid peroxidation, mitochondrial damage, and DNA single-strand breaks, which subsequently activate poly-adenosine diphosphate ribose polymerase 1 (PARP-1) and reduce cellular ATP levels. Additionally, the accumulation of vancomycin in lysosomes activates pathways that encourage apoptosis in proximal tubular cells (34).

Moreover, allergic responses such as ATIN, characterized by eosinophil infiltration, play a role in VA-AKI and often stem from delayed hypersensitivity reactions. The VTCs, which consist of vancomycin aggregates and Tamm-Horsfall glycoproteins, further contribute to kidney damage by blocking urine flow and inciting inflammation. Collectively, these mechanisms including oxidative stress, allergic responses, and the formation of VTCs, interact to induce ATN in VA-AKI, underscoring the complexity of this condition and the necessity for more research into its underlying mechanisms and potential early detection biomarkers (34).

In addition, ototoxicity, which can lead to hearing loss, is another adverse effect associated with vancomycin. The risk is particularly pronounced in patients with pre-existing hearing impairments or those receiving concurrent ototoxic medications. Monitoring for signs of hearing changes is crucial, as some effects may be irreversible. Vancomycin is associated with a rare risk of ototoxicity, particularly affecting older patients and those receiving concurrent ototoxic medications such as aminoglycosides or loop diuretics. Studies indicated that approximately 8% of patients on long-term intravenous vancomycin may experience high-frequency hearing loss (35). The underlying mechanism of drug-induced ototoxicity is believed to involve oxidative stress, leading to cochlear damage and potential hearing deficits, which can manifest as sensorineural hearing loss or tinnitus. Vancomycin ototoxicity is enhanced when co-administered with highly ototoxic antibiotics such as aminoglycosides. While many cases of ototoxicity are reversible, irreversible damage can occur, for example, when serum concentrations exceed 80 µg/mL in patients with pre-existing renal impairment (36, 37).

## 4. Resistance Mechanisms to Vancomycin

Vancomycin resistance was first identified in 1986 within Enterococci, leading to concerns about horizontal gene transfer of resistance genes to other gram-positive bacteria. Research has elucidated the genetic basis of this resistance, revealing complex mechanisms that involve multiple enzymes modifying peptidoglycan structure to evade vancomycin action. Resistance mechanisms are categorized mainly into two routes of modification, resulting in either high-level [minimum inhibitory concentration (MIC) > 64 µg/mL] or low-level (MIC 4 to 32 µg/mL) resistance, primarily related to changes in the terminal D-amino acids in Lipid II. A significant mechanism involves the replacement of D-Ala-D-Ala with D-Ala-D-Lac, resulting in a substantial decrease in vancomycin binding, conferring high resistance levels. The resistance is supported by gene clusters like vanHAX, which encode enzymes for peptidoglycan modification. Recent research has continued to reveal detailed mechanisms of vancomycin resistance, highlighting the need for ongoing study in this area (25).

**Figure 4.**
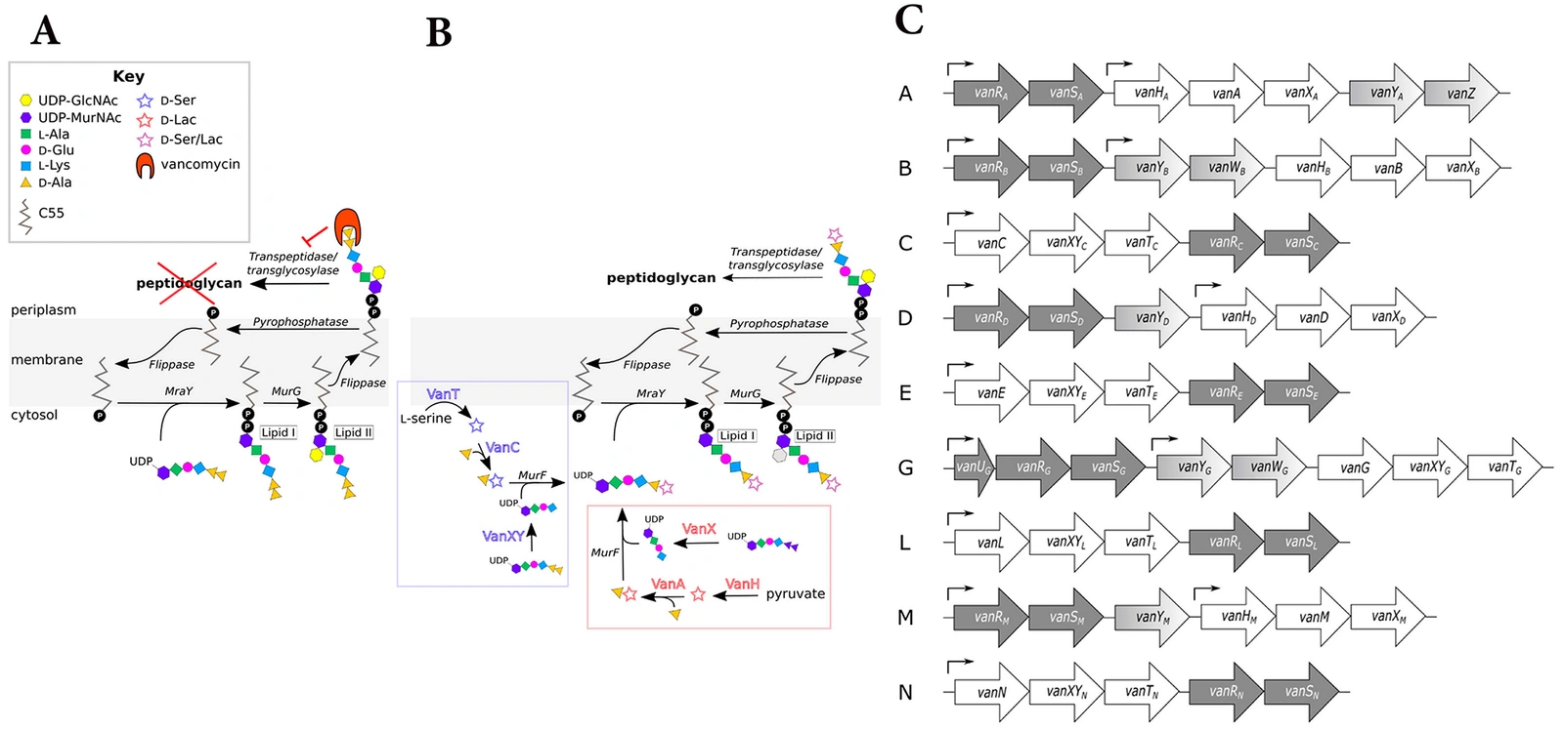
Mechanism of vancomycin resistance in Enterococci: A, in sensitive Enterococci, vancomycin attaches to the D-Ala-D-Ala terminus of the muramyl pentapeptide and disrupts the proper cross-linking of the cell wall's peptidoglycan layer; B, ithe D-Ala-D-Ala structure is altered to D-Ala-D-Ser or D-Ala-D-Lac in vancomycin-resistant Enterococci (VRE), which vancomycin cannot recognize; C, the structure of gene clusters conferring vancomycin resistance in Enterococci types A - N is outlined. Regulatory genes: Dark gray, remodeling genes: White, and accessory genes: Varying shades of gray, arrows indicate the approximate locations of promoters (39) (reprinted from an open access journal under the Creative Commons CC-BY license: https://creativecommons.org/licenses/by/4.0/).

Vancomycin-resistant bacteria (VRB) encompass several types, each exhibiting distinct resistance mechanisms. The VRE are categorized primarily into three groups: VanA, VanB, and VanC. The VanA resistance is prevalent in *E. faecalis*, *E. faecium*, and *E. casseliflavus*, characterized by high-level resistance (MIC > 64 µg/mL). In contrast, VanB resistance, mostly found in *E. faecalis*, demonstrates low-level resistance (MIC > 8 µg/mL) and retains sensitivity to teicoplanin. The VanC type is innate to *E. gallinarum*, *E. casseliflavus*, and *E. flavescens*, showing resistance with MIC values of 8 - 32 µg/mL while remaining sensitive to teicoplanin. Additionally, VanD and VanE resistances have been identified in *E. faecium* and *E. faecalis*, respectively (38). The mechanism of vancomycin resistance in Enterococci is presented in Figure 4. 

Beyond Enterococci, vancomycin-resistant Staphylococci, particularly vancomycin-resistant *Staphylococcus aureus* (VRSA), pose a significant threat, especially among MRSA with MIC values exceeding 4 µg/mL. Coagulase-negative Staphylococci, such as *S. epidermidis*, also exhibit varying levels of resistance. Additionally, certain lactic acid bacteria, including *Lactobacillus*, *Leuconostoc*, and *Pediococcus*, demonstrate intrinsic genetic resistance to vancomycin, further complicating treatment options for infections caused by these resistant strains (38).

**Figure 5.**
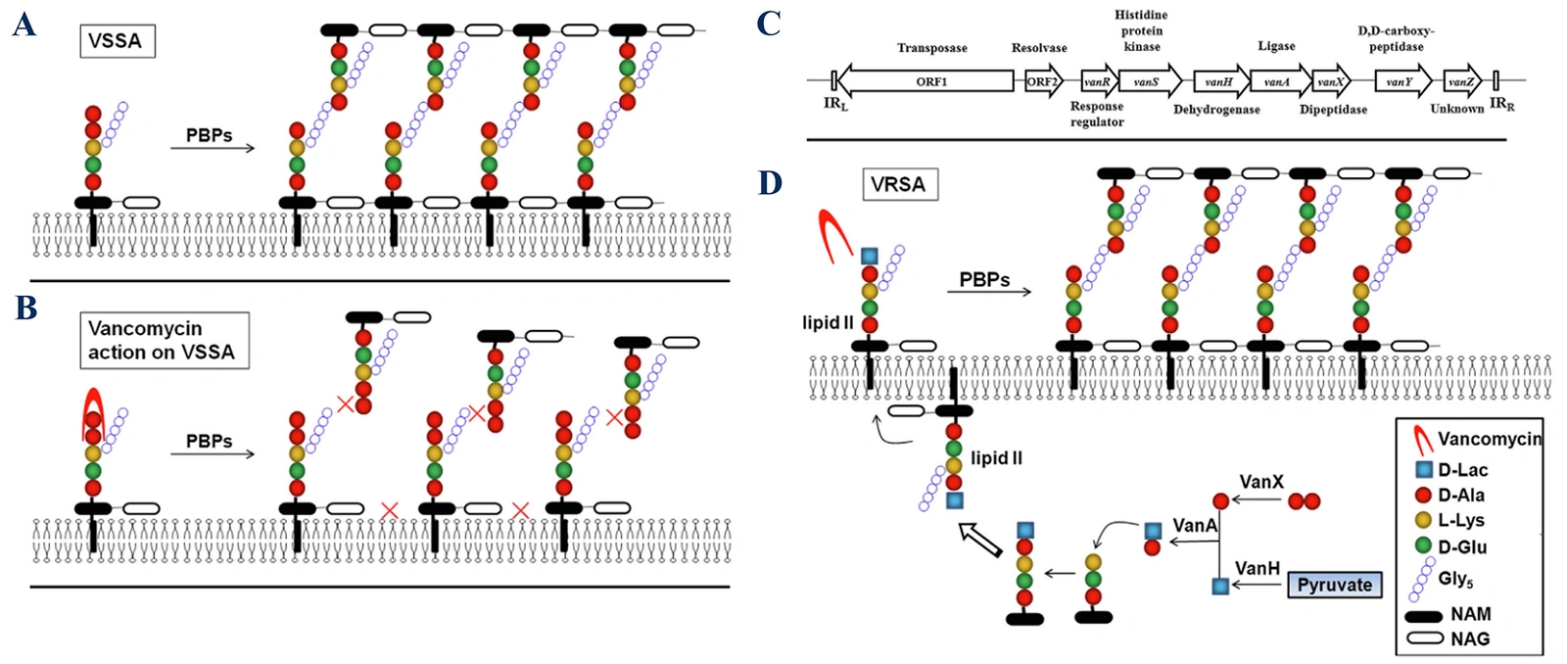
Mechanism of vancomycin resistance: A, normal peptidoglycan synthesis in vancomycin-sensitive *Staphylococcus aureus* (VSSA); B, vancomycin action on the cell wall in VSSA; C, structure of the VanA gene cluster; D, mechanism of resistance in vancomycin-resistant *Staphylococcus aureus* (VRSA) (abbreviations: D-lac, D-lactate; Gly5, Pentaglycine; NAM, N-acetylmuramic acid; and NAG, N-acetylglucosamine (44); reprinted from an open access journal under the Creative Commons CC-BY license: https://creativecommons.org/licenses/by/4.0/).

D-Ala-D-Lac-based resistance (e.g., VanA, VanB) involves replacing D-Ala-D-Ala with D-Ala-D-Lac, significantly reducing vancomycin binding. On the other hand, D-Ala-D-Ser-based resistance (e.g., VanC, VanE) involves replacing D-Ala-D-Ala with D-Ala-D-Ser, providing lower levels of resistance compared to the D-Ala-D-Lac analogue (25). Key enzymes involved in resistance include VanH, which converts pyruvate to D-lactate (40); VanA/B/D/M, the ligases that facilitate the formation of D-Ala-D-Lac; and VanC/E/G/L/N, the ligases that facilitate the formation of D-Ala-D-Ser (41). VanX is a dipeptidase that depletes D-Ala-D-Ala and enhances resistance (42), while VanS and VanR are regulatory proteins that activate resistance gene expression in response to vancomycin (39). If the D-Ala-D-Ala terminus is modified (e.g., to D-Ala-D-Lac or D-Ala-D-Ser), the affinity of vancomycin for Lipid II decreases significantly, contributing to resistance mechanisms (43). A schematic presentation of vancomycin resistance development in *S. aureus* is presented in Figure 5. 

A study presented a novel compound, VanNHdipi, developed by substituting the sugar moiety of vancomycin with a dipicolyl amine group (45). The resulting glycopeptide exhibited remarkably enhanced efficacy against VRB, showing improvements in potency up to 100-fold. The findings indicated that, like vancomycin, VanNHdipi disrupted membrane-bound processes involved in cell wall synthesis, leading to stress induction in bacteria. Furthermore, it also compromised the structural integrity of the cytoplasmic membrane, distinguishing it from the original vancomycin.

Most significantly, VanNHdipi demonstrated potent activity against critical gram-negative bacteria producing metallo-β-lactamases (MBLs), which are enzymes that confer resistance to a wide range of β-lactam antibiotics. The compound effectively inactivated various MBLs with an inhibitory concentration (IC_50_) ranging from 0.2 to 10 μM, thereby facilitating the resensitization of MBL-producing bacteria to carbapenems, a class of antibiotics often used as a last line of defense (45).

## 5. Novel Drug Delivery Systems

Poor physicochemical properties (very hydrophilic nature and high molecular weight), poor oral bioavailability, MIC creep, emergence of resistance, and a high tendency to accumulate in the kidneys are among the main issues that can be addressed using novel drug delivery systems. These systems offer many advantages to restore antibacterial activity against inducible VRE as well as VRSA. Various delivery systems such as nanoparticles (18), microparticles (23), liquid crystals (46), bioadhesives (47), self-emulsifying drug delivery systems (48), microneedles (49), hydrogels (50), and in-situ gels (23) have been investigated to enhance vancomycin's properties and effects.

Various nanoparticles based on lipid, polymer, carbon, metal, silica, and cyclodextrin have been explored for vancomycin delivery to target sites at the right time (Table 1). Among these, lipid-based nanoparticles have been extensively studied. Examples include liposomes (18), ethosomes (51), transferosomes (52), solid lipid nanoparticles (SLNs) (53), and nanostructured lipid carriers (NLCs) (54), which have been used for vancomycin passive/active delivery. For instance, to achieve a pH/lipase dual responsive release of vancomycin at the infection site, SLNs composed of ascorbyl tocopherol succinate (as a substrate of bacterial lipase), linoleic acid, and Tween 80 were prepared. The release rate of vancomycin increased in the presence of lipase enzymes and an acidic pH. Vancomycin-loaded SLNs showed an 8-fold lower MIC value for MRSA, a 2-fold higher MRSA biofilm reduction, and a 3.4-fold reduction in bacterial burden in a BALB/c mice-infected skin model compared to bare vancomycin (53).

**Table 1.** Summary of Studies on Vancomycin-Loaded/Conjugated Nanoparticles

NP Types	Investigated Antimicrobial Parameters	Microorganisms	Size (nm)	Results of Antibacterial Activity Evaluation	Ref.
**Folic acid conjugated chitosan NPs**	MIC, MBC, FIC, tolerance level, killing kinetics, inhibition zone, biofilm formation ability, bacterial cell viability, and antimicrobial mechanism	VRSA	260 ± 35	MIC, MBC, and tolerance levels of VMN were lower than those of bare VM; FIC was less than 0.5; the zone of inhibition of VMN was larger than that of bare VM; the biofilm formation ability of VRSA was reduced by 1.30% and 42.86% through treatment with bare VM and VMN, respectively; bacterial cell viability reduction for VM and VMN was 4.27% and 64.89%, respectively; without tagging with folic acid, NPs were ineffective against VRSA; VMN showed time-dependent and rapid bactericidal activity.	(16)
**Holo-transferrin conjugated PLGA NPs**	MIC	VISA and MRSA	83 ± 3	MIC of non-bioconjugated VMN was lower than that of bare VM against both MRSA and VISA. On the contrary, the MIC of holo-transferrin conjugated NPs was equal to or higher than that of free VM; the presence of holo-transferrin (the iron-saturated form of transferrin) caused bacterial growth improvement and consequently less sensitivity of bacteria.	(17)
**pH-responsive lipid (oleylamine)-polymer (chitosan) hybrid nanovesicles**	MIC, FIC, killing kinetics, antimicrobial mechanism, anti-biofilm activity, and in vivo antibacterial activity	Biofilm-forming MRSA strain	198 ± 14	VMN showed 52-fold lower MIC, higher anti-biofilm activity, faster killing rate, and 95-fold lower bacterial burden in the BALB/c mouse-infected skin model compared to bare VM; the MIC value of VMN at pH 6 was lower than that at pH 7.4; FIC was less than 0.5 up to 24 h for both pH values	(55)
**Self-assembled oleylamine grafted hyaluronic acid polymersomes**	MIC, FIC, bacterial cell viability, killing kinetics, and bacterial membrane disruption	MRSA	201 ± 3 to 361 ± 6	VMN showed a 4-fold lower MIC and faster killing rate compared to bare VM; FIC was less than 0.5; the bare VM and VMN indicated about 88.7 ± 1.2 % and 89.2 ± 0.60% dead MRSA cells, respectively; MRSA treated with bare VM showed deformed membranes, whereas MRSA treated with VMN were ruptured.	(56)
**Beta-cyclodextrin- oleylamine nanovesicles**	MIC, FIC, bacterial cell viability, killing kinetics, and bacterial membrane disruption	MRSA	125 ± 8	VMN displayed a 4-fold lower MIC and faster killing rate compared to free drug; FIC was less than 0.5; the bare VM and VMN displayed about 91.01 ± 1.48% and 92.82 ± 0.56 % dead MRSA cells, respectively; VM-treated MRSA cells displayed membrane deformation, whereas VMN-treated MRSA cells were ruptured.	(57)
**Vesicle composed of a hybrid of mPEG-b-PCL and G1-PEA dendrimers**	MIC, bacterial membrane disruption, anti-biofilm activity, killing kinetics, bacterial cell viability, and in vivo antibacterial activity	MRSA	52 ± 3	VMN displayed a 16-fold lower MIC value, higher anti-biofilm activity, faster killing rate, and a 20-fold reduction in bacterial burden in the BALB/c mice-infected skin model compared to free VM; the bare VM and VMN displayed about 98.5 ± 1.49% and 99.59 ± 0.55% dead MRSA cells.	(21)
**Sodium alginate/ polyethylene oxide blend nanofiber **	Inhibition zone and in vivo antibacterial activity	MRSA	201 ± 67	The inhibition zone diameter for VMN and VM solution was almost the same, indicating that the incorporation of VM into nanofibers did not compromise the intrinsic antibacterial activity of drug; in the case of VMN, the percentages of bacterial count in the rat-infected skin abrasion model after 48 and 72 h of treatment were significantly less than those of VM solution.	(20)
**Liposomes**	-	-	188 ± 3	VMN had a longer half-life (2.2 h) compared to the aqueous solution of VM (1.4 h); decreased accumulation in kidneys was observed for liposomal VM.	(58)
**Liposomes**	MIC, MBC, anti-biofilm activity, and in vitro resistance study	h-VISA and biofilm-forming MRSA strain	141 ± 3 to 353 ± 4	VMN showed lower MIC and MBC values for MRSA, h-VISA, and biofilms compared to VM solution; MRSA strain was not able to develop resistance against liposomal VM.	(59)
**Sterosomes**	MIC, killing kinetics, anti-biofilm activity, bacterial membrane disruption, in vivo antibacterial activity	Biofilm-forming MRSA strain	114 ± 1	VMN had less MIC, superior biofilm reduction, and a faster bacterial killing rate compared to bare VM; using the BALB/c mice-infected skin model, significant MRSA eradication was observed for VMN; VMN displayed superiority in the destruction of the MRSA cell membrane compared to bare VM.	(60)
** Niosomes **	MIC, MBC, and anti-biofilm activity	MRSA	201	VMN reduced MIC and MBC values by 2-4-fold in comparison to bare VM; VMN had a higher ability for biofilm inhibition and eradication compared to VM.	(61)
**VCM-functionalized gold/silver NPs**	MIC	MRSA	11 ± 4	VM-functionalized silver NPs showed lower MIC compared to VM-functionalized gold NPs, indicating its greater antibacterial activity.	(22)
**VCM conjugated graphene oxide NPs**	Killing kinetics, inhibition zone, anti-biofilm activity, SOD/ ROS activity of VRSA, and bacterial cell viability	VRSA	-	The inhibition zone of VMN was significantly higher than that of graphene oxide NPs or VM; a faster killing rate was observed for VMN compared to bare VM; VMN was more successful in inhibiting growth and colonization in biofilm compared to graphene oxide NPs or VM alone; VMN decreased the motility of VRSA by inducing oxidative stress; the percentage of viable bacterial cells for VMN treatment was significantly less than that of graphene oxide NPs or VM.	(62)

Abbreviations: NPs, nanoparticles; MIC, minimum inhibitory concentration; MBC, minimum bactericidal concentration; VRSA, vancomycin-resistant *Staphylococcus aureus*; VM, vancomycin; VMN, vancomycin nano-system; PLGA, poly(lactic-co-glycolic acid); VISA: vancomycin-intermediate *Staphylococcus aureus*; MRSA, methicillin-resistant *Staphylococcus aureus*; h-VISA, heteroresistant vancomycin-intermediate *Staphylococcus aureus*; SOD, superoxide dismutase; ROS, reactive oxygen species.

Using polyethylene glycol (PEG) decorated liposomes is a common method for enhancing circulation time in lipid-based nanocarriers (63). Given that antimicrobial activity is concentration/time-dependent or a combination of both, the extended blood circulation time of antimicrobial agents may enhance antimicrobial potency, thereby avoiding repeated or high-dose administration (64). FU002 is a derivative of vancomycin in which a polycationic peptide is coupled to vancomycin using a bifunctional linker. Despite its improved antibacterial activity, this derivative quickly accumulates in the liver and suffers from a short blood circulation time. To tackle these issues, liposomal encapsulation was investigated.

**Figure 6.**
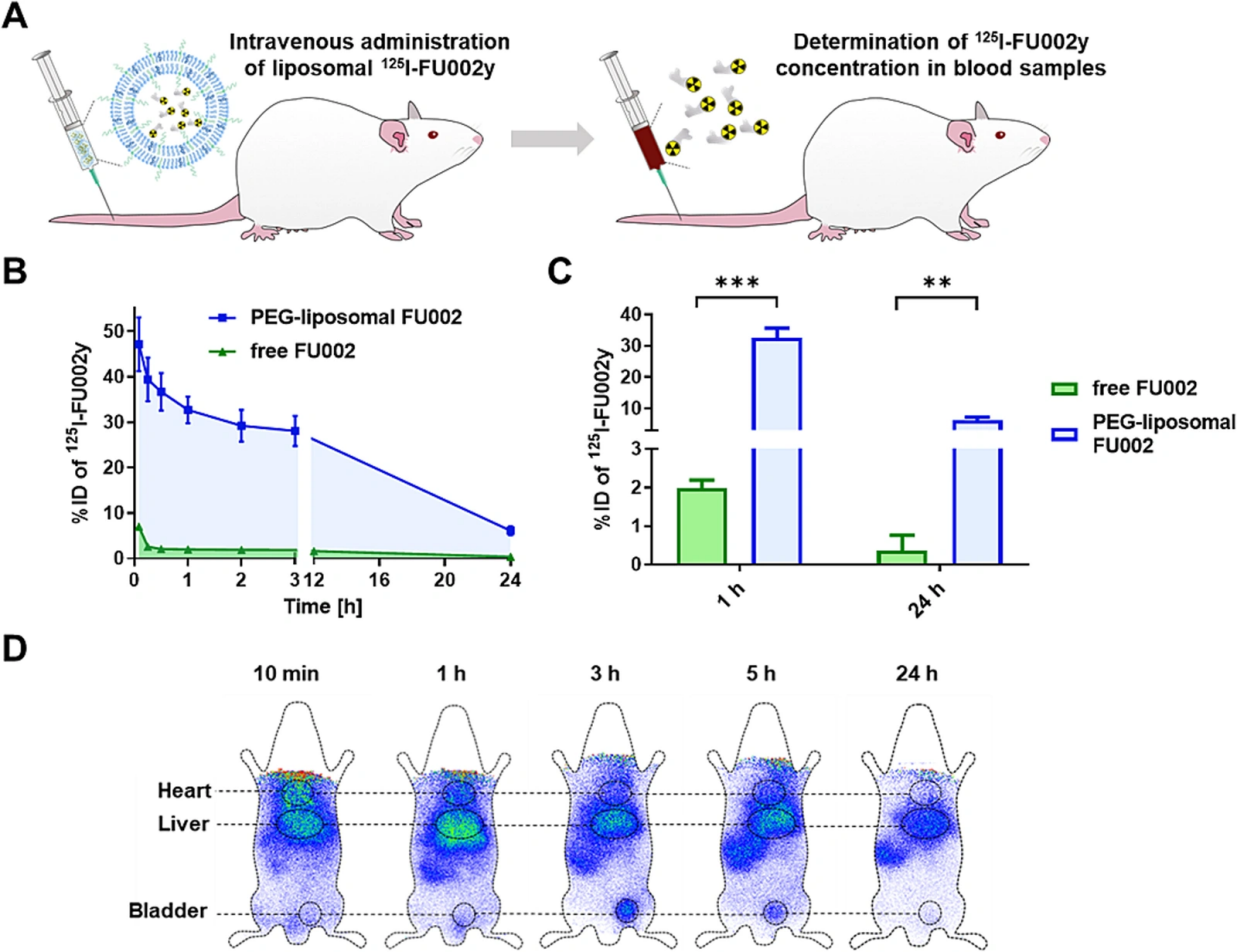
Pharmacokinetic and biodistribution studies of FU002-loaded PEGylated liposomes: A, intravenous injection of liposomal^125^I-FU002y in Wistar rats; B, time course of liposomal ^125^I-FU002y blood levels (blue) compared to free ^125^I-FU002y (green); C, compared to free ^125^I-FU002y (green), a 16-fold increase in blood levels of ^125^I-FU002y for the liposomal formulation (blue) was observed for 1 h and 24 h post-injection; D, scintigraphic images were acquired at five time points after intravenous injection of liposomal ^125^I-FU002y (64) (reprinted from an open access journal under the Creative Commons CC-BY license: https://creativecommons.org/licenses/by/4.0/). Statistically analyzed with unpaired *t*-test: ** P ≤ 0.01, *** P ≤ 0.001.

Compared to free FU002, the benefits of FU002-loaded PEGylated liposomes included increased blood circulation time following intravenous injection in Wistar rats (Figures 6A, B, and C) and increased survival rate of larvae in the *Galleria mellonella* larvae infection model. Similar to free FU002, encapsulated FU002 mainly targeted the liver (Figure 6D). Additionally, the in vitro antimicrobial activity evaluation using MRSA and VRE showed the same MIC values for free and encapsulated FU002, indicating that liposomization did not change the antimicrobial activity of FU002. Furthermore, both FU002 and liposomal FU002 broke the resistance in all three tested VRE strains (64).

The hybrid liposomal nanosystems contribute to the enhanced efficacy of antibiotics for the treatment of persistent infections. Vancomycin-loaded nucleic acid nanogels caged inside liposomes have shown a considerable decrease in both *S. aureus* intracellular and extracellular infections. The high antibiotic loading, due to the high binding affinity between a cationic cargo (vancomycin) and polyanionic DNA nanostructures, on-demand and potentiated release of vancomycin in response to lipase enzymes, and significant anti-inflammatory activity are other benefits of such hybrid liposomal nanosystems (65).

Vancomycin is used as a reducing and capping agent to form metal nanoparticles due to its glycoside structure (22, 66). To achieve an alternative approach against VRE strains, vancomycin-modified copper sulfide (CuS) nanoparticles were applied as a photothermal transducer for antimicrobial photothermal therapy. Due to the production of localized heat, photothermal therapy itself is a killing method for resistant bacteria. The CuS nanoparticles showed specific binding affinity to VRE pathogens. However, this high affinity did not imply an efficient MIC value, so vancomycin-modified CuS nanoparticles did not have a lower MIC compared to bare vancomycin. The in vivo application of these nanoparticles along with near-infrared irradiation caused elevated localized temperature, leading to approximately complete infection eradication after two days (66).

In addition to metal nanoparticles, carbon-based nanoparticles also have the potential to be used for the photokilling of resistant bacteria. Vancomycin-conjugated graphene oxide nanocomposite containing phthalocyanine (as a photosensitizer) was successfully synthesized. The nanocomposite exhibited considerable photothermal/photodynamic therapy effects, resulting in 2 to 3 logs of bacterial reduction in the in vitro photokilling effect against VRE. Moreover, the nanocomposite along with irradiation accelerated VRE-infected wound healing in BALB/C mice (67).

Increasing the poor oral bioavailability of vancomycin is another area that requires attention from researchers. Due to its high hydrophilicity and molecular weight, vancomycin suffers from poor epithelial permeability. Mesoporous silica nanoparticles have been successfully investigated to enhance the permeability of vancomycin in a Caco-2 cell model. The vancomycin-loaded surface-engineered mesoporous silica nanoparticles showed the ability to transiently open the tight junctions of the epithelial cell monolayer and were introduced as a potential delivery system for the oral administration of vancomycin (68).

The cationic leciplex, a phospholipid-based vesicular system, has demonstrated the potential to increase the oral bioavailability of vancomycin. The area under the curve (AUC) and maximum blood concentration (C_max_) of vancomycin increased about 3.41 and 2.99 times, respectively, following oral administration of lipoplex compared to the aqueous solution. The capability of lipoplex to enhance the intestinal permeability of vancomycin was attributed to its positive charge (which facilitated the adhesion of the nanocarrier onto the negatively charged surfaces of epithelial cells) and a particle size of less than 100 nm (which increased transmembrane permeability) (69).

In addition to mesoporous silica nanoparticles and leciplex, other nano-delivery systems, including poly(lactic-co-glycolic acid) (PLGA) nanoparticles (70) and surface-modified liposomes (71), have also been investigated to enhance the oral bioavailability of vancomycin (Table 2). 

**Table 2.** Summary of Some Studies on the Improvement of Vancomycin Oral Bioavailability

Delivery Systems	Investigated Permeation/Pharmacokinetic Parameters	Size (nm)	Results of Permeability/Pharmacokinetic Evaluation	Ref.
**Mesoporous silica NPs**	Apparent permeability (P_app_) and TEER values using the Caco-2 cell model	230 ± 79 to 273 ± 49	VMN showed a higher P_app_ (1.716 × 10^-5^ cm/s) compared to VM solution (0.304 × 10^-5^ cm/s); a decrease of TEER was observed for VMN, indicating the ability of VMN to temporarily open tight junctions.	(68)
**Cationic leciplex**	P_app_ using a non-everted intestinal sac model, and C_max_, T_max_, AUC, and MRT following oral administration	52.74 ± 0.91	Papp values of VMN and VM solution were 0.2240 cm/h and 0.0097 cm/h, respectively; C_max_, AUC, and MRT were higher for VMN, and T_max_ was higher for the VM solution.	(69)
**PLGA NPs**	Effective permeability coefficients (P_eff_) using in situ permeation studies	450 ± 35.29 to 466 ± 38.80	For example, the P_eff_ values were 15.75 × 10^5^ cm/s and 2.54 × 10^5^ cm/s for VMN and bare VM, respectively, at a concentration of 400 µg/mL.	(70)
**Surface-modified liposome (for VM derivative FU002)**	Caco-2 binding assay and AUC following oral administration	Approximately in the range of 100 to 150	VMN had strong binding to Caco-2 cells; AUC (%ID of ^125^I-FU002y vs. time) of VMN was about 5 times higher than bare VM.	(71)
**Self-emulsifying drug delivery systems**	Permeated VM using a mucus diffusion study and ex vivo permeation study	15.89 ± 0.30	VMN showed a higher permeation than VM across the mucous layer after 4 hours; the permeation of VMN through the intestinal mucosa was 4 - 8 times more than that of VM solution.	(48)

Abbreviations: NPs, nanoparticles; TEER, trans-epithelial electrical resistance; VM, vancomycin; VMN, vancomycin nano-system; C_max_, maximum blood concentration; T_max_, time to peak drug concentration; AUC, area under the curve; MRT, mean residence time; PLGA, poly(lactic-co-glycolic acid); ID, injected dose.

In addition to nanotechnology, other technologies also hold promise for solving vancomycin delivery issues. For instance, self-emulsifying drug delivery systems have been considered promising tools for the oral delivery of vancomycin (48) (Table 2). For vancomycin ophthalmic delivery, in addition to polymeric and lipid nanoparticles (54, 59, 72), other delivery systems such as bioadhesive minitablets (47), microemulsions (73), and thermoresponsive hydrogels (50) have been investigated.

In the case of topical/local vancomycin delivery, polymeric microparticles incorporated into injectable thermosensitive hydrogels (74), in situ forming gels and microparticles (23), microneedle arrays (49, 75), and hydrogel dressing (76) have shown promise.

## 6. Conclusions

Vancomycin has long been considered the "last resort" antibiotic for treating complicated and resistant infections caused by gram-positive bacteria. In this review, we discussed the mechanisms of action, resistance mechanisms, and toxicity associated with vancomycin. Vancomycin targets the Lipid II component and inhibits the cross-linking of peptidoglycan strands, leading to a defective bacterial cell wall structure. Over the last four decades, resistance with life-threatening consequences has emerged, involving modification of the Lipid II component, resulting in reduced vancomycin affinity. Additionally, vancomycin exhibits various systemic toxicities, with renal toxicity being the most significant.

To overcome vancomycin resistance and toxicity, the production of new, safe, and effective antibiotic drugs may be a potential solution, although it is time-consuming and costly. Another promising strategy presented in this manuscript involves using carriers to mitigate this antibiotic's adverse effects and resistance. Advanced drug delivery systems can address vancomycin-related issues by protecting the antibiotic from bacterial inactivation, enabling controlled and on-demand drug delivery, increasing on-target bioavailability, minimizing off-target accumulation, enhancing membrane permeability, and improving drug-bacteria interaction.

Furthermore, we summarized a range of novel drug delivery systems that have been investigated for enhancing the efficacy of vancomycin. These include polymeric nanoparticles, liposomes, SLNs, NLCs, nanofibers, dendrimers, metal nanoparticles, microparticles, and hydrogels. Each of these delivery systems offers unique advantages in terms of targeted delivery, which are crucial for overcoming treatment challenges. However, additional preclinical and clinical studies are necessary to bring new formulations to market. These studies should emphasize the development of more target-selective vancomycin to minimize side effects while assessing its efficacy in infected animal models. Additionally, in-depth studies on the mechanisms by which nanoparticles overcome resistance and enhance drug efficacy may pave the way for designing more effective systems.

## Data Availability

The dataset presented in the study is available on request from the corresponding author during submission or after publication.
